# Arterial hypertension assessment in a population with chronic myeloid leukemia

**DOI:** 10.1038/s41598-021-94127-2

**Published:** 2021-07-19

**Authors:** Ricardo Roa-Chamorro, Fernando Jaén-Águila, José Manuel Puerta-Puerta, Lucía Torres-Quintero, Pablo González-Bustos, Juan Diego Mediavilla-García

**Affiliations:** 1 Vascular Risk Unit, Internal Medicine, Virgen de las Nieves Hospital, Granada, Spain; 2 Hematology, Virgen de las Nieves Hospital, Granada, Spain; 3 Cardiology, Virgen de las Nieves Hospital, Granada, Spain

**Keywords:** Haematological cancer, Risk factors

## Abstract

Treatment of chronic myeloid leukaemia (CML) is based on tyrosine kinase inhibitors (TKI), whose introduction in 2001 improved the survival rate after 5 years from 40 to 90%. The longevity increase has been accompanied by a higher incidence of cardiovascular events (CVE) that can be explained due to the sum of cardiovascular risk factors (CVRF) together with the secondary effects of the TKI. The effect of the TKI over the blood pressure control is still unknown. An observational cross-sectional study of patients with CML under treatment with TKI (imatinib, dasatinib and nilotinib) was conducted. Blood pressure was analyzed through sphygmomanometer and 24-h ambulatory blood pressure monitoring (ABPM). A total of 73 patients were included, 57 treated with a single line of treatment. 32.9% of the total of individuals under this study showed uncontrolled blood pressure according to the ABPM. The factors related to uncontrolled BP were overweight, dyslipidemia, alcohol use, pulse wave velocity a high/very high cardiovascular risk. The subjects who received treatment with nilotinib did present worse control of their blood pressure in ABPM than those treated with imatinib and dasatinib (p = 0.041). This finding could indicate that an uncontrolled blood pressure is implied in the pro-inflammatory and pro-atherogenic mechanism underlying the development of the cardiovascular disease in those patients under treatment with nilotinib. The ABPM is a useful tool in the diagnosis and treatment of HT, being the reason why it should be included in the assessment of patients with CML whose HT diagnosis proves uncertain.

## Introduction

Chronic myeloid leukaemia (CML) is a myeloproliferative neoplasia caused by the clonal proliferation of a tyrosine kinase BCR-ABL-1. It was the first neoplasia treated successfully with the inhibitors of the tyrosine kinase (TKI), especially with imatinib^1^. With this drug, survival after 5 years in patients newly diagnosed with CML increased from 40 to 90%, with a life expectancy similar to healthy controls paired to age^2^. Second generation TKI, initially indicated for cases resistant to imatinib, showed subsequently an induction towards a quicker and deeper molecular response. For this reason, nilotinib and dasatinib were also indicated as first line treatment^3–5^. Bosutinib (second generation TKI) and ponatinib (third generation TKI) were introduced afterwards, being the latter designed specifically to be combined with BCR-ABL, having a very high potential to inhibit a wide range of mutations that confer resistance against other TKI (including the T315I)^6^.

The increase in survival rate in CML patients with TKI came associated with an increase in CVE. The risk of developing a CVE in patients with CML is determined by an aggregation of the classic CVRF and the secondary effects of the TKI^7–10^. In a recent study by Shallis et al., it was concluded that the average age in the diagnose of this population was 59 years^11^. It has been observed in other studies^12,13^ that CVRF frequently coexist in this age range. Besides, the TKI have been related directly with the production of metabolic secondary effects (nilotinib and dyslipidemia), as well as the development of cardiovascular disease (ponatinib and peripheral arterial occlusive disease). All this has led current guidelines^14^ to recommend adapting the choice of TKI to the comorbidities of the patient^15^. It is therefore essential to know the CVRF and the total cardiovascular risk (CVR) of the subjects even before initiating the treatment.

Regarding hypertension (HT) as a CVRF in patients with CML, in the existing literature there only exists clear evidence regarding the use of ponatinib^16^, although there are few data about the prevalence of the CVRF^12,13,17^ in the different TKI. Uncontrolled HT can pose a higher risk of CVE. ABPM allows an accurate diagnose and classification of BP, as well as the diagnose of the white coat HT, masked HT and blood pressure pattern. Patients with masked HT present a higher incidence of target organ damage than those without hypertension.

## Material and methods

Observational study of patients diagnosed with CML under active treatment with TKI corresponding to the University Hospital Virgen de las Nieves in Granada, Spain. Out of the 74 subjects diagnosed in the province, 73 accepted to participate in the study.

ITK's selection was made as follows: In elderly patients or patients with a short life expectancy, patients with many comorbidities or highly polymedicated, the first choice for TKI was imatinib. In young patients or those with high desires for future discontinuation, the first choice was a second generation TKI (2GTKI). The choice between a 2GTKI or another in first line was based on patient's comorbidities. The use of nilotinib was avoided in patients with established CV events or large CVRF. Dasatinib was avoid in patients with a history of pleural effusions or manifest fluid retention. To date, the use of bosutinib in the first line for CML in Spain is not authorized.

The patients were evaluated in a specific cardiovascular risk consultation, in which the cardiovascular history, blood and urine analysis, electrocardiogram, ankle-brachial index, pulse wave velocity and 24-h ABPM was analysed. The study was approved by the Ethical Committee of Investigation for the Province of Granada (Spain) (verification code 5c07f325e14f4a852ee4d0047025daf9baaa41c7). All patients signed the informed consent before participating in the project. All methods were performed in accordance with the relevant guidelines and regulation. The data collection was carried out between May 2018 and May 2019.

Blood pressure measurement in consultation was performed according to the recommendations of the European Society of Hypertension using a validated oscillometric blood pressure monitor (OMRON M3 IT—HEM-7131U-E)^18^. In order to carry out the ABPM, the oscillometric model SpaceLabs 90207 (SpaceLabs Inc., EEUU)^19^ was chosen. The normality values used were the latest recommended by the *ESC/ESH Guidelines for the management of arterial hypertension (2018)*^20^. Uncontrolled blood pressure (in patients without clinical arterial hypertension and with arterial hypertension under treatment) was defined as ABPM ≥ 130/80 mmHg. The ankle-brachial index was calculated through the Microlife WatchBP Office ABI system^21^, considering as pathological those quotients ≤ 0.9. For the assessment of the arterial stiffness it was performed the indirect measurement of the pulse wave velocity (PWV), using the validated system Mobil-O-Graph^22^. The reference values were determined by normalised charts estimated according to age, height, weight and central blood pressure. Regarding the target organ damage, it was considered microalbuminuria if the ratio albumin/creatine was higher than 30 mg/g in two measurements^23^. The assessment of left ventricular hypertrophy was carried out following the electrocardiographic criteria of Sokolow-Lyon^24^ and Cornell^25^.

For the comparative analysis among TKI, patients who had treatment changes during the evolution of the disease were removed from the study, classifying the patients in a first group formed by subjects who had been treated with imatinib and dasatinib and a second group determined by those treated with nilotinib.

### Ethics approval

This project was approved by the Provincial Ethics Committee of Granada, Spain.

## Results

A population of 73 patients diagnosed with CML and under active treatment with TKI was analysed. 39 patients were treated in first line with imatinib 400 mg daily, 24 with nilotinib 300 mg twice daily and 10 with dasatinib 100 mg daily. 16 patients required a change to a second line of treatment (9 nilotinib 300 mg or 400 mg twice daily, 7 dasatinib) and one to a third line with dasatinib. Mean age was 56.47 ± 15.19 years. The average duration of the treatment was 74.04 ± 54 months. 46 of the subjects were male (63.01%). The complete description of the cardiovascular risk factors appears in Table 1. The parameters refer to patients under TKI treatment. A total of 47.9% of the patients were hypertensive, with an average Systolic Blood Pressure (SBP) of 137.44 ± 19.92 mmHg and Diastolic (DBP) of 81.15 ± 10.09. All patients except one underwent 24-h ABPM. 32.9% of this total population presented hypertension in ABPM: 83.6% of them with dipper pattern, 13.7% with non-dipper pattern and 1.4% with riser pattern; 17.8% with white coat HT and 6.8% with masked HT. Out of the total of previously diagnosed hypertension, 71.4% of the subjects presented BP > 130/80 mmHg in ABPM.

**Table 1:** Prevalence of CVRF and cardiovascular disease in the population under treatment with TKI.

Item	Prevalence (%)
Sex (men)	46 (63.01%)
Age	56.07 ± 15.19
Overweight	37 (50.68%)
Obesity	19 (26.03%)
Hypertension	35 (47.95%)
Diabetes mellitus	13 (17.33%)
Dyslipidemia	40 (40.79%)
Smoking	16 (21.92%)
Alcohol	11 (15.07%)
Chronic kidney disease (GF CKD-EPI < 60 ml/min)	14 (19.18%)
Ischemic stroke	3 (4.10%)
Ischemic heart disease	3 (4.10%)
Peripheral arterial disease	6 (8.22%)
Cardiovascular disease	11 (11.07%)

Prevalence of CVRF and cardiovascular disease in subjects analyzed with CML and treatment with TKI.

*CVRF* Cardiovascular risk factors, *GF CKD-EPI* Glomerular filtration Chronic Kidney Disease Epidemiology Collaboration.

The factors related to hypertension according to ABPM were analysed. In the group with arterial hypertension in ABPM there existed significant differences regarding age, overweight, dyslipidemia, pulse wave velocity, established cardiovascular disease and high/very high cardiovascular risk. No relationship was observed between TKI and HT, although it was identified a statistical trend with nilotinib (Table 2).

**Table 2:** Comparison of CVRF, target organ damage and cardiovascular disease between subjects with controlled and uncontrolled blood pressure according ABPM.

	Controlled 48 (66.7)	Uncontrolled 24 (33.3)	p
Sex (men)	30 (62.5)	16 (66.7)	0.729
Age (mean ± sd)	54.0 ± 15.5	62.4 ± 12.5	**0.024**
BMI (Me[IQR])	26.7 [24.5–30.7]	28.4 [26.6–30.1]	0.070
Overweight	20 (41.7)	17 (70.8)	**0.020**
Obesity	12 (25)	6 (25)	1
Diabetes mellitus	8 (16.7)	5 (20.8)	0.749
Dyslipidemia	20 (41.7)	19 (79.2)	0.003
Smoking	9 (18.8)	7 (29.2)	0.316
Alcohol	5 (10.4)	6 (25)	0.163
Family story CVRF	22 (45.8)	10 (41.7)	0.737
LVH	2 (4.2)	4 (16.7)	0.091
Microalbuminuria	8 (16.7)	6 (25)	0.529
Pathological PWV	8 (16.7)	13 (56.5)	**0.001**
ABI < 0.9	13 (27.1)	7 (30.4)	0.769
Chronic kidney disease (GF CKD-EPI < 60 ml/min)	7 (14.6)	7 (29.2)	0.206
Ischemic stroke	1 (2.1)	2 (8.3)	0.256
Ischemic heart disease	2 (4.2)	1 (4.2)	1
Peripheral arterial disease	2 (4.2)	4 (16.7)	0.091
Cardiovascular disease	4 (8.3)	7 (29.2)	**0.034**
**CV risk**			**0.031**
Low	29 (60.4)	7 (29.2)	
Intermediate	8 (16.7)	5 (20.8)	
High/very high	11 (22.9)	12 (50)	
Imatinib	26 (54.2)	12 (50)	0.738
Nilotinib	18 (37.5)	14 (58.3)	0.094
Dasatinib	13 (27.1)	5 (20.8)	0.564

Values highlighted in bold underline statistically significant results.

Prevalence of CVRF, target organ injury, and cardiovascular disease in the group with controlled blood pressure control and uncontrolled blood pressure.

*ABI* ankle-brachial index, *ABPM* Ambulatory blood pressure monitoring, *BMI* body mass index, *CV* cardiovascular, *CVRF* cardiovascular risk factor, *GF CKD-EPI* Glomerular filtration Chronic Kidney Disease Epidemiology Collaboration, *LVH* Left ventricular hypertrophy, *PWV* pulse wave velocity.

A multivariate analysis was carried out, considering those variables with a p < 0.20. The variables which had influence over ABPM ≥ 130/80 mmHg were overweight, dyslipidemia, alcohol use, high pulse wave velocity and high cardiovascular risk (Table 3).

**Table 3:** Multivariate analysis: factors related to uncontrolled blood pressure according ABPM.

	OR	CI 95% to OR		Sig.
		Lower	Higher	
Overweight	7.343	1.491	36.157	0.14
Dyslipidemia	5.693	1.295	25.017	**0.021**
Alcohol	6.300	1.015	39.121	**0.048**
Pathological PWV	10.517	2.252	49.124	**0.003**
Intermediate CV risk	4.536	0.607	33.864	0.140
High CV risk	5.978	1.100	32.491	**0.048**

Values highlighted in bold underline statistically significant results.

Multivariate analysis of the factors related to uncontrolled blood pressure.

*ABPM* ambulatory blood pressure monitoring, CV cardiovascular, *OR* odds ratio, *Sig* signification, *PWV* pulse wave velocity.

For a deeper analysis of the factors related to blood pressure control and TKI, it was decided to only include those patients who had received a single drug during their oncological history. Out of the 73 initial patients, the sample was reduced to 57: imatinib 26 (45.6%), nilotinib 22 (38.6%) and dasatinib 9 (15.8%). They were divided into two groups: a first group with patients who had been treated with imatinib and dasatinib^26^ (61.40%) and a second one with patients who had received nilotinib^21^ (38.60%). The average duration of the treatment was 85.31 ± 56.58 months in the first group and 42.73 ± 33.66 months in the second. Most of the patients developed hypertension after starting treatment (69.2%). Specifically, by treatment groups, 53.85% of the patients in the imatinib + dasatinib treatment group developed hypertension after the initiation of TKI treatment, while in the nilotinib group, the number increased to 84.62% of the patients. There were no statistically significant differences between the onset of TKI and the development of hypertension between the two groups, although there was a statistical trend. 96.15% of hypertensive patients were taking antihypertensive drugs. The mean number of drugs was 1.35 ± 0.98 drugs per patient. Table 4 shows the distribution by antihypertensive pharmacological groups. No statistically significant differences were found between the number and class of antihypertensive drugs and treatment groups according to TKI. The mean values of clinical blood pressure and 24-h ABPM are gathered in Table 5. Statistically significant differences were found between the values of clinical diastolic blood pressure (p = 0.038), systolic blood pressure in the 24 h period (p = 0.017) and systolic blood pressure in the daytime period (p = 0.022). It was observed that 45.5% of the patients in treatment exclusively with nilotinib showed hypertension in ABPM (values ≥ 130/80 mmHg) against a 20% of those treated with imatinib/dasatinib, with significant statistical differences (p = 0.041) (Table 6).

**Table 4:** Pharmacological therapy for hypertension.

	Hypertension patients 26 (%)	Imatinib + dasatinib group 13 (%)	Nilotinib group 13 (%)	p
ACE inhibitors/ARBs	19 (73.07%)	8 (61.54)	11 (84.62)	0.378
Beta-blockers	7 (26.92%)	5 (38.46)	2 (15.38)	0.378
Calcium antagonists	7 (26.92%)	3 (23.08)	4 (30.77)	1
Diuretics	6 (23.07%)	3 (23.08)	3 (23.08)	1

Antihypertensive drugs used in patients with HT. They are distinguished by TKI groups (imatinib + dasatinib vs. nilotinib).

*ACE* angiotensin-converting enzyme, *ARB* angiotensin receptor blocker.

**Table 5:** Mean 24-h ABPM and clinical blood pressure values in the imatinib, dasatinib and nilotinib groups.

	Imatinib^25^	Dasatinib^9^	Imatinib + dasatinib^26^	Nilotinib^21^	(Imatinib + dasatinib vs nilotinib) p
**Clinical blood pressure**					
sBP	133.08 ± 19.82	133.44 ± 15.53	133.17 ± 18.59	138.59 ± 14.81	0.253
dBP	76.58 ± 8.38	80.44 ± 6.77	77.57 ± 8.09	82.36 ± 8.56	**0.038**
HR	73.50 ± 10.79	73.89 ± 11.40	73.60 ± 10.78	75.23 ± 13.42	0.616
**ABPM 24 h**					
24 h					
sBP	118.92 ± 9.62	120.33 ± 10.15	119.29 ± 9.62	129.00 ± 16.27	**0.017**
dBP	69.23 ± 6.78	72.44 ± 5	70.06 ± 6.46	73.45 ± 9.93	0.163
HR	69.46 ± 9.14	72.56 ± 9.36	70.26 ± 9.16	71.50 ± 12.24	0.664
Diurnal					
sBP	122.31 ± 10.11	125.00 ± 10.67	123.00 ± 10.17	132.41 ± 16.49	**0.022**
dBP	71.58 ± 7.54	76.11 ± 5.67	72.74 ± 7.31	76.82 ± 10.87	0.130
HR	71.54 ± 10.18	73.78 ± 9.13	72.11 ± 9.84	72.23 ± 10.13	0.967
Nocturnal					
sBP	111.23 ± 10.60	108.56 ± 10.38	110.54 ± 10.46	116.82 ± 16.55	0.084
dBP	62.58 ± 6.72	63.78 ± 4.78	62.89 ± 6.23	65.59 ± 8.83	0.181
HR	64.19 ± 8	68 ± 9.90	65.17 ± 8.54	65.64 ± 9.90	0.851
Dominant ABPM pattern (%)	Dipper (84%)	Dipper (100%)	Dipper (88.57%)	Dipper (90.90%)	0.617

Values highlighted in bold underline statistically significant results.

Mean 24-h ABPM and clinical blood pressure values are observed for groups of patients. Only the data of patients who have been with a single antineoplastic were collected.

The ABPM values are collected referring to the mean blood pressure values during 24 h, the mean values in the daytime period and the mean values in the night period. Data are compared between the imatinib + dasatinib and nilotinib treatment groups.

*ABPM* ambulatory blood pressure monitoring, *CML* chronic myeloid leukemia, *dBP* diastolic blood pressure, *HR* heart rate, *sBP* systolic blood pressure, *TKI* tirosin kinase inhibitors.

**Table 6:** Relationship between hypertension, white coat hypertension, masked hypertension, and uncontrolled blood pressure with the various TKI.

**Arterial hypertension**	
Imatinib	p = 0.743
Dasatinib	p = 0.732
Nilotinib	p = 0.305
Imatinib/dasatinib vs. nilotinib	p = 0.105
**White coat hypertension**	
Imatinib	p = 0.973
Dasatinib	p = 0.073
Nilotinib	p = 0.940
Imatinib/dasatinib vs. nilotinib	p = 1
**Masked hypertension**	
Imatinib	p = 0.363
Dasatinib	p = 1
Nilotinib	p = 1
Imatinib/dasatinib vs. nilotinib	p = 1
**Uncontrolled blood pressure**	
Imatinib	p = 0.738
Dasatinib	p = 0.564
Nilotinib	p = 0.094
Imatinib/dasatinib vs. nilotinib	**p = 0.041**

Values highlighted in bold underline statistically significant results.

Comparative analysis of hypertension, white coat hypertension, masked hypertension, and uncontrolled blood pressure with the groups of patients analyzed (imatinib, dasatinib, nilotinib, imatinib + dasatinib).

## Discussion

With the development of the different generations of tyrosine kinase inhibitors, CML has entirely changed its prognosis, evolving from a fatal disease in a few months to a chronic pathology, with a life expectancy similar to that of the general population^27^. The increase in survival rate has been accompanied by a higher incidence of cardiovascular disease, produced by the presence of CVRF and cardiometabolic toxic effects of the very TKI. The cardiovascular toxic effects produced by the TKI can be explained due to the fact that the inhibition of the tyrosine kinase BCR-ABL1 is not selective, thus being able to also affect other kinases, including those which act on the vascular biology^10^, such as the receptors of the vascular endothelial growth factor (VEGF) 1 to 3, TIE-2, receptors A and B derived from platelet growth (PDGFRAα/β) and fibroblast growth factor receptors (FGFR) 1 to 4^28^.

Knowledge of the TKI cardiovascular profile is paramount in order to attain the most favourable treatment for these patients. Imatinib presents a favourable metabolic and vascular profile. Although at the beginning of its development it was suggested that it could cause cardiomyopathy, this suspicion was never confirmed afterwards. It has been observed a lower incidence of CVD with imatinib compared to nilotinib^8^. Dasatinib presents a non-haematological safety profile similar to imatinib, except for the development of pleural effusion^29^. There are contradictory data regarding the production of pulmonary hypertension^30,31^. Nilotinib has been related to the development of dyslipidemia, coronary disease, cerebrovascular disease and peripheral arterial occlusive disease. It has not been related to thromboembolic disease^7,32^. No cardiovascular complications with bosutinib have been described. Ponatinib is related to the apparition of hypertension, venous thromboembolic and arterial disease, which can rapidly develop after exposure to the drug^26,33^.

Regarding HT as a cardiovascular risk factor in patients with CML, it has only been clearly related to the use of ponatinib^16^. It has been described that ponatinib inhibits VEGFR, therefore producing a decrease of the angiogenesis, vascular rarefaction, lower production of nitric oxide and an increase in endothelin. No increase of HT incidence has been described in patients treated with imatinib, dasatinib or nilotinib.

As per our study, the population included has a mean age and male sex prevalence similar to that described in other series of patients with CML^11,12^. In Spain, the prevalence of HT in the adult population fluctuates between 33.3% and 42.6%^34^, 13.8% of diabetes mellitus^35^, 50.5% dyslipidemia^36^, 39.3% overweight and 21.6% obesity^37^. The percentage of patients with high blood pressure and overweight in our population is higher than the national average, with similar results with respect to the rest of CVRF.

Regarding the factors associated to hypertension in ABPM, they are similar to those of the general population^38–40^, being statistically significant the results for age (uncontrolled blood pressure patients are significantly older), overweight (although not obesity), dyslipidemia, alcohol use, pulse wave velocity (whose result increases directly with age and blood pressure values) and established cardiovascular disease. Besides, patients with uncontrolled blood pressure had a higher cardiovascular risk. The long-term analysis of the ENESTnd (Evaluating Nilotinib Efficacy and Safety in Clinical Trials-Newly Diagnosed Patients) study revealed that the CVR calculation according to the Framingham Risk Score predicted the development of CVE during treatment with nilotinib^41,42^. These data, together with the results of our study, would make us carry out a stricter monitoring and tighter control of the CVRF in those patients with worse blood pressure control.

Regarding the analysis in order to evaluate the relationship between arterial hypertension in ABPM and TKI, it was decided to remove the patients who had been treated with two or more types of TKI during the evolution of the illness. Out of the 73 initial patients, the sample was reduced to 57. Most patients developed hypertension after initiating treatment with TKI (69.2%). Although there were no significant differences between the two groups, there was a trend in the nilotinib group. There were no differences between the number and class of antihypertensive drugs in the two groups. The statistical analysis showed a trend towards relevance in the prevalence of hypertension and ABPM (BP ≥ 130/80 mmHg) in those patients under treatment with nilotinib compared to those who were not. Statistically significant differences were found between the values of clinical diastolic blood pressure, systolic blood pressure in the 24 h period and systolic blood pressure in the daytime period. Patients in treatment with nilotinib presented worse blood pressure control in ABPM than the patients in the group with imatinib/dasatinib, showing a higher incidence of cardiovascular events.

Nilotinib produces dyslipidemia in 50% of the subjects treated, besides potentially generating ischemic cardiopathy and cerebrovascular disease. However, the mechanism through which nilotinib produces CVE is not well known. It has been observed that the patients treated with nilotinib present a higher inflammatory state than the patients treated with imatinib and dasatinib, due to an increased level of low density lipoproteins (c-LDL), which could lead to a pro-oxidative and pro-inflammatory state^43^ with higher levels of inflammatory cytokines, TNF-alpha and IL-6, as well as an increase in expression of proatherogenic endothelial adhesion molecules (E-selectin, VCAM-1 ICAM-1), which could contribute to the atherosclerotic process. The results of our study, in which patients treated with nilotinib presented a worse control of blood pressure in comparison with those treated with imatinib and dasatinib, make us consider the hypothesis that hypertension could also be involved in the development of the atherosclerotic mechanism affecting those patients treated with nilotinib^44^.

We are aware of the limitations of our work since it consists of an observational uncontrolled study. Besides, we are dealing with a rare disease, which determines the small size of the sample. Nevertheless, this is a pioneering study in the field of cardiovascular risk in CML, and we believe that it serves as a reference for further studies.

## Conclusions

As far as we know, this is the first study analysing clinical BP and 24-h ABPM in patients with CML treated with TKI. It has been observed that our population presents a higher prevalence of HT than the national average of the general population. Besides, those patients treated with nilotinib showed a higher percentage of patients with uncontrolled blood pressure than the group with imatinib and dasatinib. This leads us to consider the hypothesis that uncontrolled hypertension could be involved in the pro-atherogenic mechanism of nilotinib underlying the development of cardiovascular disease in those patients. 24-h ABPM has proved useful in order to improve the diagnose and treatment of HT in patients under TKI, especially for those treated with nilotinib and ponatinib. It can also help clarifying the pro-atherogenic mechanisms of the TKI.

## Data Availability

All data collected are available upon request to the corresponding author.
